# Dyslipidemia Management in the Middle East: Recommendations for Overcoming Challenges and Enhancing Outcomes With Pitavastatin

**DOI:** 10.7759/cureus.110155

**Published:** 2026-06-02

**Authors:** Ahmad J AlSarraf, Zuhier Awan, Osama Baghal, Hamid Bayeh, Said M Chaaban, Mustafa T Jammal, Roland Kassab, Tony Abdelmassih, Osama Okkeh, Kausik Ray, Hassan El-Tamimi, Mohamad Iskandarani

**Affiliations:** 1 Cardiology, Sabah Al-Ahmad Cardiac Centre, Al-Amiri Hospital, Kuwait City, KWT; 2 Clinical Biochemistry, King Abdulaziz University Faculty of Medicine, Jeddah, SAU; 3 Cardiology, Al Khalidi Medical Center, Amman, JOR; 4 Cardiology, Notre Dame des Secours University Hospital, Byblos, LBN; 5 Interventional Cardiology, Beirut Arab University, Beirut, LBN; 6 Interventional Cardiology, Private Practice, Amman, JOR; 7 Cardiology, Hôtel-Dieu de France Hospital, Saint Joseph University of Beirut, Beirut, LBN; 8 Primary Care and Public Health, Imperial College London, London, GBR; 9 Cardiology, Mediclinic Parkview Hospital, Dubai, ARE; 10 Medical Affairs, Algorithm, Beirut, LBN

**Keywords:** cardiovascular protection, dyslipidemia, ldl-c, middle east, pitavastatin

## Abstract

Pitavastatin, indicated for lowering low-density lipoprotein cholesterol (LDL-C), is particularly beneficial for patients who experience side effects with other statins. When used in combination with other cholesterol-lowering medications, such as ezetimibe, pitavastatin can enhance LDL-C reduction, improving patient outcomes and facilitating achievement of LDL-C targets. This paper reviews the available literature on the use of pitavastatin for cardiovascular (CV) protection in patients with dyslipidemia, drawing on both published evidence and insights from lipidologists and cardiologists who participated in an advisory board. It highlights pitavastatin’s effectiveness in managing dyslipidemia and its potential to mitigate CV complications, particularly in patients with atherosclerotic cardiovascular disease (ASCVD). Feedback from practicing healthcare providers across several countries in the Middle East, including Kuwait, Saudi Arabia, the United Arab Emirates, Lebanon, and Jordan, emphasizes the importance of early intervention and achieving optimal LDL-C levels to reduce CV risk. It also underscores the relevance of adhering to international guidelines for dyslipidemia management in regional clinical practice.

This paper offers practical recommendations to address challenges and improve the management of dyslipidemia in the Middle East through the use of pitavastatin, with the aim of achieving LDL-C targets and reducing CV events.

## Introduction and background

Atherosclerotic cardiovascular diseases (ASCVDs) are among the leading causes of death worldwide [1]. The accumulation of cholesterol in the arterial wall throughout the life course contributes to atherosclerosis, which results from the interplay of multiple risk factors, including genetic and environmental factors, in addition to dyslipidemia [2]. The prevalence of dyslipidemia has increased alarmingly in the Middle East over the past two decades, contributing to a corresponding rise in CVDs [3]. The prevalence of ASCVD is estimated at 20.9%, posing a significant socioeconomic burden on the region [4]. Current international guidelines, including those from the European Society of Cardiology/European Atherosclerosis Society (ESC/EAS) and the American College of Cardiology/American Heart Association (ACC/AHA), advocate low-density lipoprotein cholesterol (LDL-C) lowering as a primary therapeutic target to reduce ASCVD risk [5,6].

Lipid-lowering therapies (LLTs), particularly statins, which remain the gold standard for the treatment of dyslipidemia, have demonstrated significant benefits in reducing ASCVD risk [7-10]. Pitavastatin, one of the newer-generation statins, not only provides significant LDL-C reduction but also contributes to lowering CV risk in eligible patients [11]. With its favorable safety profile, it represents an effective option for patients requiring moderate-intensity statin therapy and may help improve treatment adherence.

This paper is a narrative review that summarizes the association between dyslipidemia and ASCVD, highlighting the benefits of LLTs, with a particular focus on pitavastatin. Additionally, it presents insights from lipidologists and cardiologists from the Middle East who participated in an advisory board meeting to discuss the use of pitavastatin for CV protection in patients with dyslipidemia. This paper also offers recommendations for the timely management of dyslipidemia using pitavastatin to help prevent or mitigate ASCVD.

## Review

Atherosclerosis pathophysiology and risk factors

Atherosclerosis results from the interplay of genetic factors, environmental influences, risk factors, and ageing. It develops through the gradual accumulation of cholesterol within the arterial walls, leading to the formation of atherosclerotic plaques, narrowing and stiffening of the arteries, impaired blood flow, and ultimately heart attacks and strokes [2]. Several risk factors, including diabetes, hypertension, and smoking, cause endothelial injury and promote cholesterol accumulation within blood vessels, leading to atherosclerotic plaque formation. Around 60% of individuals worldwide experience CV events, including heart attacks and strokes, by the age of 54 years. Individuals with multiple risk factors are at a higher risk of developing CVD [2]. However, most CV events occur in individuals who do not have extremely high levels of risk factors [12], underscoring the importance of screening, primary prevention, and early treatment in people with CV risk factors. Cholesterol is transported in apolipoprotein B (ApoB)-containing lipoproteins [13]. Genetic predisposition, cumulative cholesterol exposure, and time are major contributors to the development of atherosclerosis. Genetic susceptibility helps explain differences in lipid profiles among individuals with similar lifestyles, as well as the occurrence of CV events in patients with relatively few risk factors [14]. Multiple gene mutations contribute to dyslipidemia by affecting lipid transport, synthesis, or clearance [15]. Mutations in the low-density lipoprotein receptor (LDLR), ApoB, and proprotein convertase subtilisin/kexin type 9 (PCSK9) genes are commonly evaluated in familial hypercholesterolemia, while lipoprotein lipase (LPL) mutations are associated with familial hypertriglyceridemia [15]. Identifying such mutations is critical for the diagnosis, management, and treatment of inherited lipid disorders [15].

Dyslipidemia management

CV Risk and Treatment Targets

LLT is essential for reducing elevated cholesterol levels, particularly LDL-C, to prevent ASCVD. The risk of atherosclerotic events (CV risk) is directly proportional to LDL-C levels and cumulative exposure to elevated LDL-C concentrations [1,16,17]. Treatment goals for LDL-C are stratified according to CV risk [16,18]. High- and very high-risk patients, such as those with ASCVD, require more aggressive LDL-C-lowering strategies, whereas lower-risk patients may benefit primarily from lifestyle modifications, with pharmacological therapy guided by changes in risk status over time [16,18]. According to the European Society of Cardiology (ESC) and the European Atherosclerosis Society (EAS), the primary treatment goals are LDL-C levels below 100 mg/dL for moderate-risk patients in primary prevention, below 70 mg/dL for high-risk patients, and below 55 mg/dL for very high-risk patients, together with at least a 50% reduction from baseline in both primary and secondary prevention settings [16,19]. Early initiation of treatment may reduce the need for more intensive therapeutic interventions at a later stage [16].

Pitavastatin

LDL-C-lowering therapies primarily aim to increase the number of LDL-C receptors and maintain their function in the liver, thereby enhancing cholesterol clearance from the bloodstream [20]. Proprotein convertase subtilisin/kexin type 9 (PCSK9) inhibitors help preserve receptor activity [21], while dietary modifications and ezetimibe reduce cholesterol absorption, prompting the liver to increase receptor expression [22]. Statins and bempedoic acid reduce endogenous cholesterol synthesis, which also leads to increased LDL-C receptor expression [23].

Statins constitute the first-line treatment and remain the most widely prescribed LLTs [1]. Pitavastatin is one of the newer-generation statins and was approved by the US Food and Drug Administration (FDA) in 2009 and by the European Medicines Agency (EMA) in 2010 for the treatment of dyslipidemia and the prevention of CVD. While high-intensity statins, such as atorvastatin and rosuvastatin, are generally considered for high-risk patients, pitavastatin is classified as a moderate- to high-intensity statin [24]. A dose of 4 mg has been shown to produce LDL-C reductions comparable to those achieved with atorvastatin 20-40 mg or rosuvastatin 10-20 mg, supporting personalized treatment strategies to achieve lipid goals [25]. Pitavastatin is often used in patients with moderately elevated LDL-C levels or in those who experience side effects with high-intensity statins. It has demonstrated similar or greater efficacy in improving lipid parameters and achieving LDL-C targets compared with other statins [11] (Table 1). Pitavastatin reduces total cholesterol by up to 39%, LDL-C by up to 50%, and triglycerides by up to 30%, while increasing high-density lipoprotein cholesterol (HDL-C) levels by up to 10% [26,27]. Although not currently approved at this therapeutic dose, pitavastatin 16 mg/day was reported in a large meta-analysis to be more potent in reducing LDL-C than rosuvastatin and fluvastatin [28]. Pitavastatin has a high bioavailability (50%), allowing effective treatment at lower doses (1, 2, or 4 mg) compared with atorvastatin (10, 20, 40, or 80 mg) or rosuvastatin (5, 10, 20, or 40 mg), which may improve patient adherence [29]. A network meta-analysis of 50 randomized controlled trials involving patients with dyslipidemia, diabetes, or ASCVD showed that rosuvastatin had the greatest LDL-C-lowering effect, followed by atorvastatin and pitavastatin [30]. Furthermore, switching from other statins, including pravastatin, simvastatin, fluvastatin, and atorvastatin, to pitavastatin was associated with increases in serum HDL-C levels of 21.0%, 11.8%, 20.1%, and 15.8%, respectively [31].

**Table 1:** Characteristics of commonly used statins CV: cardiovascular, CVD: cardiovascular disease, HDL-C: high-density lipoprotein cholesterol, LDL-C: low-density lipoprotein cholesterol, MACE: major adverse cardiovascular events, MI: myocardial infarction.

Statin	Typical dose range (mg/day)	LDL-C reduction	HDL-C increase	TG reduction	CV outcome evidence
Atorvastatin (C₃₃H₃₅FN₂O₅) [17]	10-80	35%-60%	5%-15%	20%-30%	Significant reduction in MI and stroke [32]. Reduced mortality and MACE [33]. Effective for both primary and secondary prevention [34]
Fluvastatin (C₂₄H₂₆FNO₄) [35]	20-80	20%-35%	2%-5%	10%-20%	Reduced MACE [36]. Mild effect on mortality and stroke
Lovastatin (C₂₄H₃₆O₅) [37]	20-80	20%-40%	4%-10%	10%-20%	Reduced MACE and mortality [38]. Less potent than newer statins
Simvastatin (C₂₅H₃₈O) [39]	10-40 (max 80 with caution)	30%-50%	5%-10%	15%-30%	Significant reduction in total mortality, heart attack, and stroke [40]. Proven benefit in secondary prevention [38]
Pravastatin (C₂₃H₃₅NaO₇) [41]	10-40	20%-34%	5%-10%	10%-20%	Reduced CV mortality and non-fatal MI [42]. Beneficial for primary prevention [43]
Rosuvastatin (C₂₂H₂₈FN₃O₆S) [44]	5-40	45%-63%	8%-15%	20%-30%	Significant reduction in MI, stroke, and CVD mortality [7]. Highly effective for both primary and secondary prevention [10]
Pitavastatin (C₅₀H₄₆CaF₂N₂O₈) [45]	1–4	31%-50%	5%-10%	15%-30%	Fewer MACE in emerging studies [8,9,46]. LDL-C reduction comparable to other statins, with lower risk of drug interactions

Pitavastatin use in clinical settings

Monotherapy

**Figure 1:**
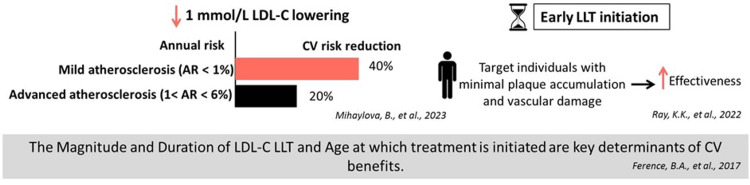
LDL-C lowering and CV risk reduction AR: annual risk, CV: cardiovascular, LDL-C: low-density lipoprotein cholesterol, LLT: lipid-lowering therapy. Source: This figure was created by the authors based on the data from Ray et al. [1], Mihaylova et al. [48], and Ference et al. [49], and was generated using Microsoft PowerPoint (Microsoft Corp., Redmond, WA, USA), with one graph exported from Microsoft Excel and some icons from BioRender (BioRender, Toronto, ON, Canada).

Statins reduce LDL-C levels and prevent CV events (Table 1). Data from both statin and non-statin trials indicate that the CV benefits are proportional to the absolute reduction in LDL-C, with similar outcomes observed for every 1 mmol/L decrease in LDL-C levels, regardless of the therapy used [47]. A 1 mmol/L reduction in LDL-C lowers the rate of CV events by up to 40% in individuals with mild atherosclerosis, compared with 20% in those with advanced atherosclerosis [48] (Figure 1). Early initiation of LLTs is associated with greater effectiveness, as these therapies target individuals with minimal plaque accumulation and vascular damage [1] (Figure 1). Therefore, the magnitude and duration of LDL-C lowering, as well as the age at which treatment is initiated, are key determinants of CV benefit [49] (Figure 1). A 50% reduction in LDL-C initiated at the age of 60 years is associated with a 27% reduction in CV risk; however, initiating the same intervention 15 years earlier provides substantially greater lifetime benefit [1,49].

A meta-analysis of six studies by Katsiki et al. showed that pitavastatin use was associated with improved vascular endothelial function, significantly increasing flow-mediated dilation (FMD) (WMD = 2.45%) [50]. In patients with carotid intima-media thickness (CIMT) > 1.1 mm and LDL-C levels > 100 mg/dL, intensive pitavastatin treatment resulted in a reduction in CIMT of 0.024 mm after 12 months [51]. Beyond significantly reducing LDL-C and non-HDL-C levels, pitavastatin was associated with a significant reduction in coronary atherosclerotic plaque volume in patients with acute coronary syndrome (ACS), with decreases of 16.9 ± 13.9% and 18.1 ± 14.2% observed in coronary arteries 10 months after treatment initiation [52]. Among patients with stable coronary artery disease (CAD), pitavastatin 4 mg/day was associated with a 19% reduction in all-cause mortality, a 43% reduction in ACS, and a 14% reduction in the need for coronary revascularization [8]. In a study comparing pitavastatin (2 mg/day) and atorvastatin (10 mg/day) in 664 patients with hypercholesterolemia and high ASCVD risk, both agents demonstrated similar lipid-lowering effects. However, pitavastatin was more effective in reducing the risk of major CV events, including death, ACS, stroke, and the need for coronary revascularization [11]. Patients with diabetes receiving statin therapy showed a significant reduction in all-cause mortality over a follow-up period of up to nine years compared with patients not receiving statins. The statins ranked from greatest to least benefit were pitavastatin, rosuvastatin, pravastatin, simvastatin, atorvastatin, fluvastatin, and lovastatin [53]. In the same patient population, pitavastatin was associated with the greatest reduction in CV mortality [54].

Combination Therapy

**Figure 2:**
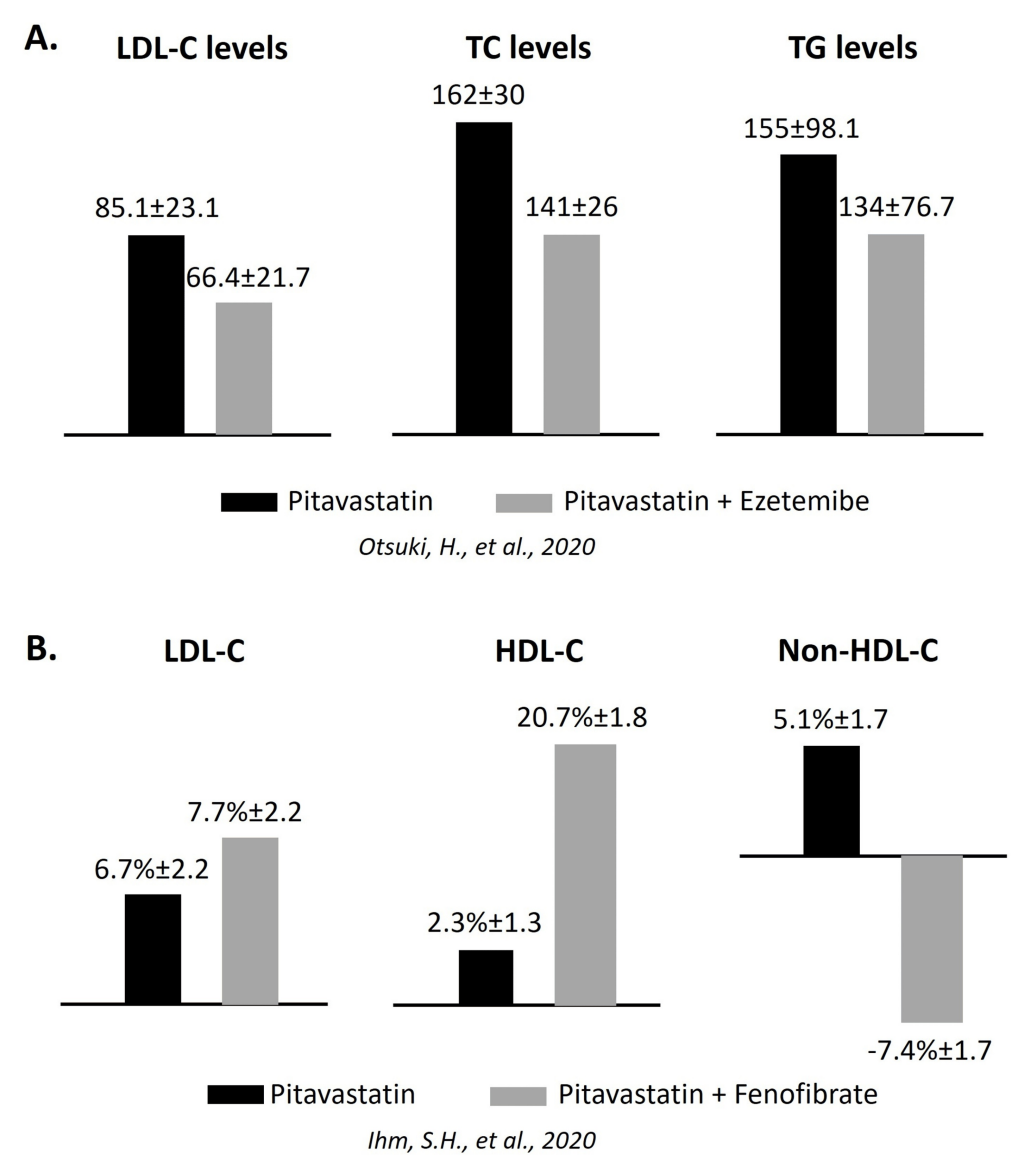
Pitavastatin efficacy: combination versus monotherapy (A) Reductions in LDL-C, TC, and TG levels in patients with a history of atherosclerosis treated with pitavastatin alone versus pitavastatin + ezetimibe [57]. (B) Percentage of change in LDL‑C, HDL-C, and non-HDL-C, after eight weeks of treatment with pitavastatin alone versus pitavastatin + fenofibrate in patients with dyslipidemia at high CVD risk [58]. CVD: cardiovascular disease, HDL-C: high-density lipoprotein cholesterol, LDL-C: low-density lipoprotein cholesterol, TC: total cholesterol, TG: triglyceride. Source: This figure was created by the authors based on the cited references and was generated using Microsoft Excel and exported to Microsoft PowerPoint (Microsoft Corp., Redmond, WA, USA).

Patients who are unlikely to achieve their lipid goals with monotherapy should be considered for combination therapy earlier in the treatment course, alongside lifestyle interventions such as dietary modifications and physical activity. Unlike many other statins, pitavastatin is predominantly metabolized by uridine glucuronyl transferases (UGTs), with only minor involvement of the CYP2C9 pathway. This distinctive metabolic profile significantly reduces the potential for drug-drug interactions, making pitavastatin particularly suitable for patients who may require combination therapy [26,55]. When combined with ezetimibe, pitavastatin has demonstrated superior LDL-C reduction. A randomized trial conducted by Jeong et al. showed that pitavastatin plus ezetimibe reduced LDL-C levels by 52.8% ± 11.2%, compared with 37.1% ± 14.1% with pitavastatin monotherapy [56]. Similar benefits were observed in patients with a history of ACS, in whom combination therapy (pitavastatin + ezetimibe) significantly outperformed pitavastatin monotherapy in reducing LDL-C levels (66.4 ± 21.7 vs 85.1 ± 23.1 mg/dL), total cholesterol levels (141 ± 26 vs 162 ± 30 mg/dL), and triglyceride levels (134 ± 76.7 vs 155 ± 98.1 mg/dL) [57] (Figure 2A). A meta-analysis of nine randomized controlled trials in patients with CAD confirmed these findings, demonstrating greater improvements in lipid parameters with the pitavastatin-ezetimibe combination than with pitavastatin alone. Furthermore, combining pitavastatin with fenofibrate in patients with dyslipidemia resulted in greater reductions in non-HDL-C and LDL-C levels, as well as greater increases in HDL-C levels, compared with pitavastatin monotherapy [58] (Figure 2B).

In addition to improving lipid parameters, the combination of pitavastatin and ezetimibe has also demonstrated prognostic benefits, reducing all-cause mortality by 55% and non-fatal stroke by 23% compared with monotherapy [57].

Safety Considerations

Statins are most commonly associated with muscle-related adverse events, such as myalgia and myopathy. However, pitavastatin, due to its unique pharmacokinetic profile and minimal metabolism via the CYP450 pathway, has been associated with a lower incidence of muscle-related adverse events compared with other statins [59]. While statin therapy has been linked to an increased risk of diabetes, the overall impact on glycaemia is minimal, with a low incidence of new-onset diabetes (approximately 1 case per 1000 treated patients) [60]. In this context, pitavastatin has been shown to be safe and to significantly reduce the cumulative incidence of type 2 diabetes, as demonstrated in the J-PREDICT study [61]. A meta-analysis of randomized controlled trials further indicated that pitavastatin did not significantly alter fasting plasma glucose or glycated hemoglobin levels and was not associated with new-onset diabetes [62]. When compared with low- to moderate-intensity atorvastatin or rosuvastatin, patients receiving pitavastatin exhibited a consistently lower risk of new-onset diabetes [63]. Additionally, pitavastatin has been associated with significant improvements in estimated glomerular filtration rate in patients with renal disease [64].

Unmet Needs and Challenges in LDL-C Management

Despite the proven efficacy of statins in lowering LDL-C levels, only a few patients achieve their LDL-C targets, even when receiving the most potent statins. The Da Vinci study (2017-2018) reported that, in Europe, only about 30% of patients reached their LDL-C targets [65]. The situation is even more concerning in high-risk patients. According to the SANTORINI study, only 20.1% of very-high-risk patients across 14 European countries achieved their LDL-C targets [66]. This is largely due to underdosing, reliance on statin monotherapy, and poor adherence.

Adherence Rate

Types and doses of statins, along with their associated side effects and dosing frequency, are closely linked to treatment adherence. Only about 50% of patients remain adherent to statin therapy at one year [67]. Among patients who had previously experienced an acute myocardial infarction, 20.2% exhibited low adherence to treatment, while 69% showed good adherence during the first year [68]. High-intensity statins provide greater LDL-C reduction at lower doses and with less frequent dosing (i.e., fewer tablets per day) compared with low- to moderate-intensity statins, which may contribute to higher adherence rates [69]. Among high-intensity statins, adherence was highest with atorvastatin, followed by simvastatin and then pravastatin [69]. Given its favorable safety and tolerability profile, patients who switched to pitavastatin showed higher treatment adherence compared with their previous statin therapy [31]. The minimal drug-drug interaction potential of pitavastatin and its low risk of new-onset diabetes may further improve adherence in patients with diabetes [60].

Collectively, clinical evidence on the efficacy and safety of pitavastatin supports improved adherence to treatment, which may translate into better long-term outcomes.

Reducing pill burden through single-pill combinations (SPCs) has been consistently associated with improved adherence, particularly in the long-term management of chronic conditions such as hypertension and dyslipidemia. In hypertension, SPCs have demonstrated superior adherence rates and better blood pressure control compared with multi-pill regimens, largely due to simplified dosing and reduced treatment complexity [70,71]. Similarly, combining a statin with ezetimibe in a single-pill formulation offers a more practical and effective approach than intensifying statin monotherapy [72]. SPCs in LLT have also been shown to enhance patient adherence and improve lipid goal attainment compared with separate-pill combinations [73,74], making them a valuable strategy, especially for patients who are far from LDL-C targets.

Non-adherence to medications is a major contributor to patient morbidity, mortality, and healthcare costs [75]. Evidence consistently shows that higher adherence to statins correlates with better LDL-C goal attainment. Among patients with CAD, around 80% were treatment-adherent, and of these, 85.8% achieved LDL-C levels < 100 mg/dL, whereas only 66% of non-adherent patients reached this level [76]. Similarly, in a secondary prevention cohort of patients with diabetes, LDL-C levels were significantly lower, and patients were approximately twice as likely (around 60% versus 30%) to achieve LDL-C targets ≤ 2.5 mmol/L when adherent compared with non-adherent patients [77]. Adherence was also associated with LDL-C reduction across all statin intensity categories. A 10% improvement in adherence was associated with LDL-C reductions of 3.5, 5.8, and 7.1 mg/dL in low-, moderate-, and high-intensity groups, respectively [78]. High adherence and treatment intensity were also strongly associated with improved CV outcomes, including a composite endpoint of CV death or hospitalization for myocardial infarction, unstable angina, ischemic stroke, heart failure, or revascularization [79]. High adherence to therapy after acute myocardial infarction was linked to a markedly lower crude mortality rate compared with low adherence (5.3% versus 20.9%) [68]. Low adherence was consistently associated with higher all-cause, CV, and non-CV mortality [68]. Patients who adhered to high-intensity statin therapy experienced the greatest CV protection, with a 40% risk reduction [79]. In contrast, those who failed to maintain adherence derived substantially less benefit, regardless of statin intensity [79]. Among patients prescribed high-intensity statins, poor adherence resulted in only modest CV benefit, comparable to that seen in non-adherent patients receiving low-intensity therapy, emphasizing that adherence, not intensity alone, is crucial for meaningful CV risk reduction [79]. Every 10% increase in a combined adherence-intensity score was associated with approximately a 10% reduction in CV event risk [79]. Tailoring statin therapy by optimally selecting the agent, dose, and regimen based on individual patient risk, safety profile, and tolerability has been shown to enhance adherence and reduce treatment discontinuation [80].

Recommendations

**Figure 3:**
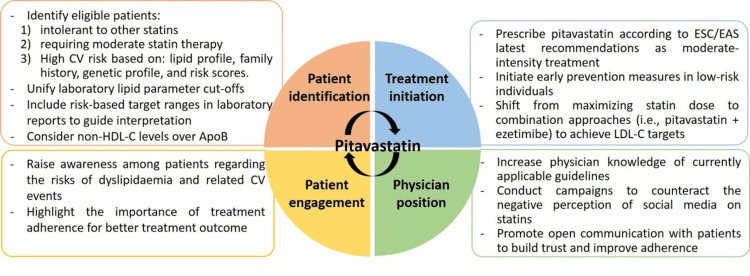
Recommendations for an optimal management of dyslipidemia in the Middle East ApoB: apolipoprotein B, CV: cardiovascular, ESC/EAS: European Society of Cardiology /European Atherosclerosis Society, HDL-C: high-density lipoprotein cholesterol, LDL-C: low-density lipoprotein cholesterol. Source: This figure was created by the authors based on the insights gathered from experts and was generated using Microsoft PowerPoint (Microsoft Corp., Redmond, WA, USA).

Against the backdrop of a rising burden of dyslipidemia and CV risk across the Middle East, a panel of regional cardiologists and lipidologists convened to provide key insights on optimizing dyslipidemia management and preventing or mitigating atherosclerosis (Figure 3). The panel addressed gaps in screening practices and identified patient profiles eligible for treatment. The experts highlighted the benefits of pitavastatin and supported its use in combination therapy over high-dose statin monotherapy. Early intervention for primary prevention was strongly advocated, with emphasis on initiating treatment before disease progression to reduce the burden of ASCVD in the region. The panel also encouraged a shift toward combination therapy rather than reliance on maximal statin doses to achieve meaningful LDL-C reduction and attainment of LDL-C targets. The role of physicians in adhering to international guidelines and providing patient education was identified as a cornerstone of optimal dyslipidemia management and CV risk reduction.

## Conclusions

Pitavastatin has emerged as a valuable alternative for patients who are intolerant to other statins. While it demonstrates strong potential in improving lipid parameters, combination therapy (particularly with agents such as ezetimibe) may be necessary in some cases to achieve LDL-C targets. Beyond lipid-lowering, pitavastatin has shown favorable effects on atherosclerosis-related characteristics, such as plaque volume and vessel wall thickness, reinforcing its potential role in CV risk reduction.

The evidence and expert perspectives presented in this paper highlight the strategic importance of pitavastatin within the evolving treatment paradigm for dyslipidemia. By aligning regional clinical practice with international guidelines and promoting patient-centered care, these recommendations offer a pragmatic framework for improving cardiovascular outcomes in the Middle East.
